# The Role of Macrophages in *Staphylococcus aureus* Infection

**DOI:** 10.3389/fimmu.2020.620339

**Published:** 2021-01-19

**Authors:** Grace R. Pidwill, Josie F. Gibson, Joby Cole, Stephen A. Renshaw, Simon J. Foster

**Affiliations:** ^1^ Department of Molecular Biology and Biotechnology, University of Sheffield, Sheffield, United Kingdom; ^2^ Florey Institute, University of Sheffield, Sheffield, United Kingdom; ^3^ The Bateson Centre, University of Sheffield, Sheffield, United Kingdom; ^4^ Department of Infection, Immunity and Cardiovascular Disease, Medical School, University of Sheffield, Sheffield, United Kingdom

**Keywords:** macrophage, *Staphylococcus*, phagocytosis, immunity, immune evasion

## Abstract

*Staphylococcus aureus* is a member of the human commensal microflora that exists, apparently benignly, at multiple sites on the host. However, as an opportunist pathogen it can also cause a range of serious diseases. This requires an ability to circumvent the innate immune system to establish an infection. Professional phagocytes, primarily macrophages and neutrophils, are key innate immune cells which interact with *S. aureus*, acting as gatekeepers to contain and resolve infection. Recent studies have highlighted the important roles of macrophages during *S. aureus* infections, using a wide array of killing mechanisms. In defense, *S. aureus* has evolved multiple strategies to survive within, manipulate and escape from macrophages, allowing them to not only subvert but also exploit this key element of our immune system. Macrophage-*S. aureus* interactions are multifaceted and have direct roles in infection outcome. In depth understanding of these host-pathogen interactions may be useful for future therapeutic developments. This review examines macrophage interactions with *S. aureus* throughout all stages of infection, with special emphasis on mechanisms that determine infection outcome.

## Introduction

*Staphylococcus aureus* is a Gram-positive commensal bacterium frequently found in the upper respiratory tract (1, 2), alongside various other locations on the human host (3). *S. aureus* is part of the normal microbiota, colonizing 40% of new-born babies and 50% of adults intermittently or permanently, normally without any ill-effects (1, 4). Despite this, *S. aureus* can become pathogenic, with colonization an important reservoir for infection (5).

Human diseases caused by *S. aureus* range from minor skin infections to life threatening bacteremia and meningitis. *S. aureus* is one of the most frequent causes of nosocomial and community-acquired pneumonia, skin and soft-tissue infections or bloodstream infections (6). Serious *S. aureus* infections cause approximately 20,000 deaths a year in the US, and 5,000 in the EU, costing an estimated €380 million in EU health costs (7, 8). A contributing factor to the high mortality rate of *S. aureus* infections is increasing antimicrobial resistance. Methicillin Resistant *Staphylococcus aureus* (MRSA) bacteremia has a high mortality rate: 30% to 40% (9–12). *S. aureus* resistance to antibiotics is widespread in both community and nosocomial-acquired infection. Some *S. aureus* strains have even developed resistance to the last-resort antibiotic vancomycin (13) and vaccine candidates have thus far been unsuccessful (14, 15). *S. aureus* infections represent a significant risk to human health, highlighting the pressing need for alternative prophylaxis and treatments.

The immune response to *S. aureus* infection is complex. Infection occurs when *S. aureus* breaches host external barriers, for example through a tissue injury. In most cases, an efficient immune response is mounted, involving innate immune cell recruitment and eventual clearance of infection. Macrophages, as antigen presenting cells, also activate the adaptive immune response. As such, phagocytes play a vital role in locating, restricting and destroying *S. aureus*.

Macrophage interactions with *S. aureus* are of particular interest. Macrophages are responsible for phagocytic uptake of the majority of invading bacteria and employ a multitude of bacterial killing mechanisms to effectively kill *S. aureus*. Despite this, some *S. aureus* are able to survive within macrophages - a source for intracellular persistence which eventually enables further bacterial dissemination (16–18). *S. aureus* can survive within mature macrophage phagosomes (16, 19, 20), as well as cause uncontrollable infection within monocyte-derived macrophages (MDMs) (18). Furthermore, *S. aureus* can evade and manipulate macrophages, using many strategies to impede macrophage recruitment, phagocytosis and degrative abilities (21–25). Understanding these complex host-pathogen interactions may provide promising new therapeutic targets, which are urgently required due to rising *S. aureus* antibiotic resistance.

**Figure 1:**
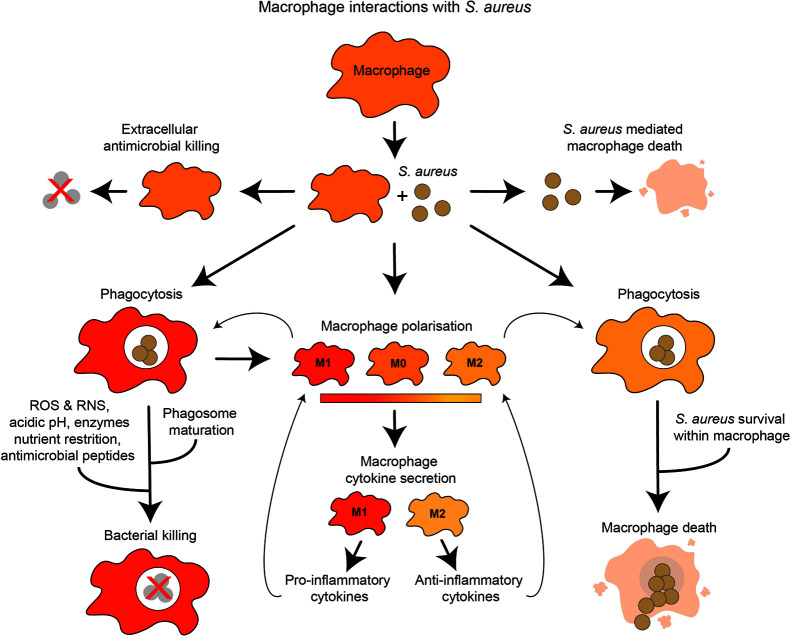
Overview of macrophage interactions with *S. aureus*. Presence of *S. aureus* influences macrophage polarization and cytokine secretion toward either pro-inflammatory or anti-inflammatory. *S. aureus* may be subject to extracellular or intracellular killing by macrophages. Macrophages may be killed by extracellular *S. aureus* factors. *S. aureus* phagocytosis by macrophages may lead to intracellular killing, which may destroy the bacteria using antimicrobial mechanisms. Alternatively, *S. aureus* may evade these mechanisms, proliferate within the macrophage and cause macrophage death.

This review examines macrophage interactions with *S. aureus*, from the role of macrophages in *S. aureus* infection dynamics to specific macrophage-*S. aureus* interactions, including macrophage recruitment, phagocytosis, macrophage polarization, bacterial killing mechanisms and nutrient restriction (**Figure 1**).

## What Are Macrophages?

Macrophages are professional phagocytes, able to engulf microorganisms and trigger responses leading to microbial death. Macrophages, like their close relatives neutrophils, both of which are professional phagocytes and are derived from myeloid precursor cells (22, 26), are an important part of the innate immune response. However, each phagocyte has multiple differences in cellular properties and functions. Both macrophages and neutrophils sense and migrate toward sites of infection and can phagocytose and kill invading pathogens. However, macrophages, as antigen presenting cells, also play a key role in activation of the adaptive immune response by presenting antigens of phagocytosed pathogens (27). Neutrophils are commonly the first immune cell to reach an infected site, and may be more bactericidal, whereas monocytes (which may differentiate into macrophages) are typically attracted later on (28). In comparison to neutrophils, macrophages are adapted to be much less reactive, which may be to avoid attacking self-antigens or stimulating unwanted immune responses due to being resident within tissues for a longer lifespan (29). Neutrophils are derived from within the bone marrow and have a very short lifespan which is thought to limit stimulation of unnecessary inflammation (28). In contrast, macrophages may live for weeks to months (30, 31) and are found within tissues through the body, termed tissue resident macrophages.

There are different cell lineage sources which give rise to tissue-resident macrophages. Traditionally, it was thought that macrophages develop only from circulating monocytes, the precursor cells for some macrophages and dendritic cells. Monocytes represent around 10% of leukocytes in humans, while tissue-resident macrophages represent another 10% to 15% (although this may increase following inflammatory stimulus) (26, 32). Monocytes develop from hematopoietic stem cells in the bone marrow (26) and circulate in the blood for 1 to 2 days, after which they die unless recruited to tissues for differentiation (26, 33, 34). However, many tissue-resident macrophages self-renew within the tissue (35). Self-renewing macrophages are derived from embryonic-origin cells which are seeded to sites of the body before birth (36–38), with examples including liver Kupffer cells, Langerhans skin cells and brain microglia (35, 39–41). Other macrophage populations develop from the macrophage and dendritic cell precursor (MDP) cell, a precursor to monocytes (42).

Once macrophages have differentiated according to their tissue, they develop distinct transcriptional profiles and are named according to tissue location (43). The properties of varied tissue-resident cells have been extensively reviewed (44, 45). However, macrophage function remains similar regardless of tissue location: (i) coordinating tissue development, (ii) tissue homeostasis through clearing apoptotic/senescent cells, (iii) acting as sentinels which survey and monitor changes in the tissue, and (iv) responding to pathogens in infection (26).

Kupffer cells are the largest group of tissue-resident macrophages in the body, making up 80% to 90% of the total population (46, 47). They display a unique phenotype characterized by downregulation of CR3, expression of liver-specific lectin CLEC4F and tissue-specific complement receptor CRIg (48, 49). Through a variety of receptors, Kupffer cells filter blood and mediate clearance of waste products and non–self-antigens (48, 50, 51). The close proximity of Kupffer cells to sinusoids also facilitates best access to pathogens arriving in the liver (46).

As mentioned above, Langerhans cells (LCs) are also self-renewing, although if they are exhausted by, for example, UV radiation, they are replaced by bone-marrow-derived precursor cells (52). LCs develop dendritic cell (DC) characteristics in the epidermis, and as such share attributes with both DCs and macrophages (53). Similar to tissue-resident macrophages, LCs self-renew and have a long half-life (approximately 2 months), however, like DCs, LCs can travel to lymph nodes (52, 53). Their presence at the barrier of the skin suggests a role as immune sentinels (54).

Macrophage diversity enables tissue-specific phenotypes which help macrophages to perform their function. However, macrophages are unified in their phagocytic and innate immune functions, allowing bridging of the innate and adaptive responses.

## The Key Role of Macrophages in *S. aureus* Infection Outcome

A wide range of diseases are caused by *S. aureus*, from minor skin infections to life-threatening diseases, for example bacteremia and endocarditis. Numerous *S. aureus* infections of humans are associated with abscess formation (55) and in murine bacteremia infection models, kidney abscess formation is a key outcome (56, 57). Macrophages have a central role in *S. aureus* infection dynamics. Murine blood infection begins with hematogenous transit of extracellular *S. aureus*, which are rapidly phagocytosed in the liver by Kupffer cells. More than 90% of *S. aureus* are sequestered by the liver (58) - the majority of bacteria are then effectively killed. A small number of bacteria can survive intracellularly, ultimately escaping to form microabcesses in the liver. Extracellular *S. aureus* may also disseminate to seed kidney abscesses (59, 60).

The importance of macrophages in *S. aureus* infection is highlighted when macrophages are depleted in animal infection models. Mice lacking macrophages have increased bacterial burden and mortality following *S. aureus* sepsis (61). Similarly, in murine airway infection, macrophages are required for clearance of *S. aureus*, since loss of alveolar macrophages inhibited killing of bacteria at 5 hpi (62), significantly enhanced mortality (63), and increased bacterial load in the lungs (64). In zebrafish, macrophages phagocytose the majority of the initial bacterial inoculum and, similar to mice, loss of macrophages leads to increased *S. aureus* susceptibility (65, 66). Phagocytes are a known intracellular niche for *S. aureus*, allowing bacterial survival and eventual escape, allowing dissemination throughout the host (67–69). Human monocyte-derived macrophages (MDMs) also permit intracellular *S. aureus* survival and bacterial escape (16, 18). Despite this, macrophages efficiently phagocytose and degrade most *S. aureus*, with just a small proportion of bacteria surviving to potentially lead to dissemination throughout the host (59). Thus, the intraphagocyte niche represents a population bottleneck for *S. aureus* (70), as demonstrated for other intracellular pathogens including *Salmonella enterica* and *Bacillus anthracis* (71, 72). Micro-abscesses in the liver are formed from surviving bacterial cells which escape from macrophages. It has been demonstrated that *S. aureus* abscesses are formed by single, or very small numbers of bacteria (69, 70), leading to the emergence of clonal populations within abscesses. Depletion of macrophages causes loss of clonality whereas depletion of neutrophils does not (59), indicating that macrophages are the key phagocyte responsible for the emergence of clonality. Kupffer cells are especially instrumental as an intraphagocyte niche leading to the emergence of clonality in *S. aureus* murine sepsis infection, largely due to their key role in filtering blood (59, 61).

Extracellular bacteria, which have escaped macrophages can also seed infection at distant sites through the bloodstream. After staphylococcal cells survive and multiply inside Kupffer cells, the bacteria can escape into the peritoneal cavity where they are phagocytosed by peritoneal macrophages, which provide another intracellular niche, promoting dissemination to peritoneal organs (60). Cycles of macrophage phagocytosis and bacterial escape can allow *S. aureus* to survive intracellularly over time (73). Although macrophages are crucial for initial infection dynamics, neutrophils are thought to be significant for dissemination. Extracellular bacteria in the bloodstream may be phagocytosed by neutrophils, which can act as Trojan horses enabling spread to other organs, including the kidneys (59, 68). Together, these studies highlight the importance of macrophages in controlling the initial bacterial sepsis inoculum specifically in restricting early infection stages, and macrophage involvement in *S. aureus* infection features, including formation of a population bottleneck, clonal abscess formation and eventual dissemination.

## Phagocytosis of *S. aureus* by Macrophages

As described above, macrophages are an important host defense against *S. aureus* infection, but in order to effectively eliminate *S. aureus*, macrophages must first locate and phagocytose the invading bacteria.

### Recruitment of Macrophages to *S. aureus* Infection Sites

Phagocyte recruitment to *S. aureus* is coordinated through responding to host immune effectors released in response to *S. aureus*, or signals derived from *S. aureus* itself. Initial host responses to *S. aureus* are initiated by cells found at infected sites, often epithelial cells at mucosal surfaces. Epithelial cells sense invading *S. aureus via* pathogen recognition receptors (PRRs) which can recognize many staphylococcal molecules, including lipoproteins, lipoteichoic acid (LTA), phenol soluble modulins, protein A, toxins, and peptidoglycan (PGN) (74). Epithelial PRR signaling leads to phagocyte recruitment and activation by inducing pro-inflammatory cytokine and chemokine production; including granulocyte-macrophage colony-stimulating factor (GM-CSF), granulocyte colony-stimulating factor (G-CSF), monocyte chemotactic protein-1 (MCP-1), macrophage inflammatory protein 3α (MIP-3α), IL-6, IL-1β, and IL-8 (75–78). Additionally, formylated peptides produced by *S. aureus* directly act as chemoattractants for macrophages (79) and *S. aureus* molecules activate the complement cascade (80), leading to release of strong phagocyte chemoattractant, C5a.

Macrophage recruitment has been demonstrated in *S. aureus* murine studies. MCP-1 is important for macrophage activation and clearance of *S. aureus* infection (81). Following *S. aureus* brain infection in mice, gene expression of multiple pro-inflammatory cytokines and chemokines are upregulated, leading to macrophage recruitment (82). In peritoneal infection, particulate *S. aureus* cell envelope promotes phagocyte recruitment by inducing chemotactic cytokine production (83). Of note, some macrophages subtypes, including Kupffer cells, are tissue-resident which may be recruited to local infection sites (84), whereas monocyte-derived macrophages are recruited to sites of infection from circulation in the blood (85). *S. aureus* also has strategies to prevent immune cell recruitment, such as expressing chemotaxis inhibitory protein of *Staphylococcus aureus* (CHIPS), which blocks phagocyte binding to activated complement proteins or formylated peptides excreted by *S. aureus* (86, 87). After recruitment to sites of infection, macrophages become activated and produce cytokines to enhance the immune response; discussed in the macrophage functional changes in response to *S. aureus* infection section.

### Phagocytosis

**Figure 2:**
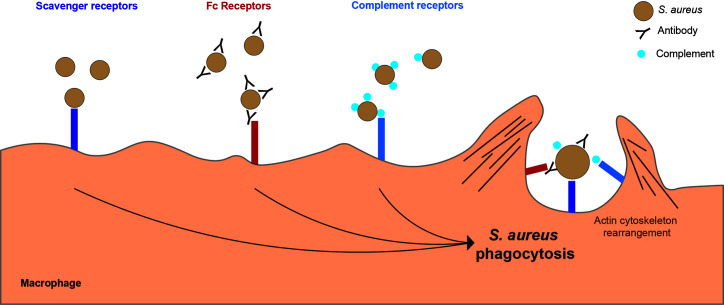
Key macrophage receptors used in phagocytosis of *S. aureus*. There are several receptors on the surface of a macrophages which can bind to *S. aureus* leading to phagocytosis. Scavenger receptors bind directly to *S. aureus*. Fc receptors bind to the Fc region of antibodies which have bound to *S. aureus*. Complement receptors bind to complement proteins which act as opsonins and are bound to *S. aureus*.

Macrophages utilize micropinocytosis, macropinocytosis, receptor-mediated endocytosis and phagocytosis to ingest particles, fluids and molecules. Micropinocytosis is used for non-specific uptake of fluid and small molecules, while macropinocytosis can non-specifically engulf larger volumes of extracellular fluid and larger particles, including bacterial cells (88–90). Receptor-mediated endocytosis is the selective uptake of macromolecules bound to surface receptors. Receptor-mediated endocytosis is clathrin-dependent, micropinocytosis can involve clathrin pathways, but clathrin is not essential (88), while phagocytosis and macropinocytosis are actin-dependent (91). Phagocytosis is receptor-mediated targeted uptake of particles larger than 0.5 µm, and represents the primary pathway used by macrophages to internalize *S. aureus* (88). The physical state of bacterial cells is important for *S. aureus* phagocytosis, with particulate rather than soluble cell wall required to stimulate an efficient phagocyte immune response (83). *S. aureus* phagocytosis events occur following engagement of multiple receptors on the macrophage surface, including scavenger receptors (SRs), complement receptors and Fc receptors (**Figure 2**). The actin cytoskeleton at the cell membrane forms a phagocytic cup which extends to surround the extracellular bacterial cells and contracts to close the cup, forming a bacteria-containing phagosome within the phagocytic cell (91).

### Scavenger Receptors

The SRs are a diverse group of receptors which recognize a wide range of pathogenic molecules, for example, proteins, polysaccharides, lipids, CpG motifs and lipoteichoic acid (LTA). SRs are grouped into classes based on what they bind, with *S. aureus* known to interact with multiple SR classes (92, 93). Macrophage SRs can bind to LTAs found on surface of Gram-positive bacteria, including *S. aureus* (94), leading to increased macrophage phagocytosis in an opsonin-independent manner (95). Scavenger receptor A (SR-A) contributes to Kupffer cell phagocytosis of *S. aureus* through mannose-binding lectin (a member of the C-type lectin family, which also binds to bacterial cells and activates the complement cascade), increasing SR-A expression on Kupffer cells (96). As well as increasing SR expression, mannose-binding lectin is also involved in opsonin-dependent *S. aureus* phagocytosis by phagocytes (97). Surfactant protein A (SP-A), like mannose-binding lectin, is a member of the C-type lectin family. Addition of SP-A to alveolar macrophages (AMs) increases *S. aureus* phagocytosis, potentially by upregulation of SR-A expression, as demonstrated for *S. pneumoniae* (98). SP-A can also act as an opsonin, binding to both *S. aureus* and SP-A receptors on macrophages. Interestingly, macrophages lacking SP-A receptors upregulate SR-A, promoting non-opsonic phagocytosis (99). Macrophage receptor with collagenous structure (MARCO) is another SR involved in macrophage phagocytosis of *S. aureus*, and is especially important in AM and Kupffer cell phagocytosis of *S. aureus* (100, 101). AMs from SR-A and MARCO knock-out mice showed reduced phagocytosis of *S. aureus* (102). Interestingly, the role of SRs in *S. aureus* infection appears to be dependent on the type of infection. Mice deficient in three different SRs (SR-A, CD36, and MARCO) were protected in peritoneal infection, but adversely effected in pulmonary infection (103). Furthermore, the importance of SRs appears to be dependent on *S. aureus* strain, with some strains showing no change in phagocytosis when SR binding is inhibited in human MDMs (104). Therefore, it is difficult to define a single role of SRs in *S. aureus* infection. However, it is clear that SRs are involved in non-opsonized *S. aureus* phagocytosis, and may play an important role in controlling lung infection.

### Complement Receptors

The complement cascade is part of the innate immune system which targets pathogens, mediated by multiple complement proteins. The key complement component is C3 which, when cleaved by C3 convertase, generates important complement effector components to mediate three main activities: pathogens can be directly targeted with the formation of a membrane attack complex to cause cell lysis, complement proteins can promote recruitment of phagocytes to the infection site and complement proteins can act as opsonins to promote phagocytosis of coated pathogens.

Multiple *S. aureus* cell surface molecules activate the complement cascade in human sera (80), with changes in complement component levels observed in patients with *S. aureus* bacteremia (105). Furthermore, human serum studies show that mannose-binding lectin promotes complement activation in response to *S. aureus* (106), while depletion of complement is detrimental in *S. aureus* murine bacteremia or septic arthritis infections (107). A mouse model of *S. aureus* septic arthritis showed that deficiency in C3 increases susceptibility to infection, potentially through decreased peritoneal macrophage phagocytosis (108). The complement components used as opsonins are C3b and iC3b, these can bind phagocyte complement receptors CR1, or CR3 and CR4, respectively. Macrophage-expressed complement receptors, CR3 and CR4, promote binding and internalization of iC3b opsonized *S. aureus* (109). A therapeutic use of antibody complexes which interact with erythrocyte CR1 and *S. aureus* have been developed leading to enhanced bacterial degradation by macrophages (110).

*S. aureus* expresses multiple virulence factors to target complement components. To inhibit complement activation, *S. aureus* secretes extracellular fibrinogen-binding protein (Efb), which binds to C3, blocking complement cascade effects including opsonization (111). To interfere with C3 convertases, *S. aureus* expresses staphylococcal complement inhibitor (SCIN) (112). Although SCIN is a human-specific virulence factor, a modified version used in animal models indicated that targeting complement is important for host adaptation (113). *S. aureus* also blocks complement opsonization. A secreted protein, *Staphylococcus aureus* binder of IgG (Sbi), has multiple functions including binding C3b, and acting to inhibit complement activation and opsonin-mediated macrophage phagocytosis (114). Similarly, the *S. aureus* protein, extracellular complement binding protein (Ecb) is used to inhibit C3b interactions with CR1 (115). Another role of complement activation is immune cell recruitment, where complement component C5a is a chemoattractant. S*. aureus* reduces phagocyte recruitment, using CHIPS, which binds to C5a (86, 87). Together, these bacterial defenses act to reduce complement-aided phagocytosis of *S. aureus*. The large number of virulence factors targeting complement highlights the importance of complement-mediated immunity against *S. aureus*.

### Fc Receptors

Fc receptors on the surfaces of phagocytes bind to the Fc region of antibodies. Invading pathogens opsonized with antibodies are more readily engulfed by phagocytes. Macrophages express Fcγ receptors, which bind IgG antibodies, triggering phagocytosis (116–118). Antibodies against *S. aureus* are detected in human sera in both healthy individuals and patients with *S. aureus* infection (119). There are specific IgG antibodies against staphylococcal *α*-*hemolysin* in the human population which are present from a young age and increase in prevalence during infection (120). There are differences in IgG antibody levels present dependent on *S. aureus* colonization of individuals, with colonization associated with higher IgG antibody titers (121).

*S. aureus* expresses virulence factors which inhibit antibody-mediated phagocytosis. Protein A (SpA) and Sbi interact with the Fc region of human IgG antibodies (122, 123). This inhibits the normal ability of the Fc region of IgG to bind to Fc receptors on phagocyte membranes, which has been thought to hide *S. aureus* from antibody-mediated phagocytosis. Despite this, it has been demonstrated that *S. aureus* strains with more protein A, and therefore more bound IgG, were not phagocytosed less by alveolar macrophages in mice (124). Furthermore, phagocytosis by neutrophils was actually higher for clinical strains with greater IgG binding than for commensal strains (125). These unexpected results could be due to differences between strains, or may be due to the lack of significant changes in the rate of phagocytosis caused by opsonin (126), suggesting that antibody opsonization is not essential for adequate *S. aureus* phagocytosis. Another virulence factor *S. aureus* uses to target antibodies is staphylokinase (SAK), which triggers degradation of IgG, as well as C3b on the bacterial cell surface (127). Since *S. aureus* has multiple strategies to target antibodies, it is likely beneficial for the bacteria to inhibit antibody binding, although whether this is to specifically protect against antibody mediated-phagocytosis is unclear.

Collectively, the presence of scavenger, complement and Fc receptors gives phagocytes their unique phagocytic capabilities. For example, if an Fc receptor is expressed on a non-phagocytic cell, that cell gains the ability to phagocytose in a similar manner to phagocytes (128). Many studies examine individual receptors in isolation to simplify their characterization, however it is important to note that in reality, all these receptors work simultaneously together to coordinate phagocytic engulfment of targets, including *S. aureus*.

## Macrophage Functional Changes in Response to *S. aureus*

Upon interaction with *S. aureus*, macrophages may become activated and create a positive immune response to control infection, for example, by promoting phagocytosis and releasing pro-inflammatory cytokines. However, in some cases macrophage responses may be manipulated by *S. aureus*, leading to ineffective or even detrimental host responses (**Figure 1**). Macrophages respond to stimuli such as cytokines in their local environment which alter macrophage functions. Under homeostatic conditions, tissue macrophages are efficient at tissue repair and healing, often characterized as ‘M2’, with increased arginase metabolism (129, 130). In response to danger, for example infection, macrophages can become pro-inflammatory and efficient at pathogen killing, often characterized as ‘M1’ with enhanced nitric oxide (NO) production (130).

The M1 (pro-inflammatory) and M2 (anti-inflammatory) macrophage classifications are used widely in research and are referred to in this text. However, it is important to note that the M1 and M2 characterizations are based on *in vitro* studies which hypothetically represent two points on a continuum upon which macrophages lie. Furthermore, M1 and M2 definitions have been inaccurately associated with classical and alternatively polarized macrophages, respectively (131). These *in vitro* descriptions may not always correlate to *in vivo* macrophage phenotypes, which varies dependent on cell origin and microenvironment, and multiple stimuli in the *in vivo* environment may change over time, for example during infection progression (132).

**Figure 3:**
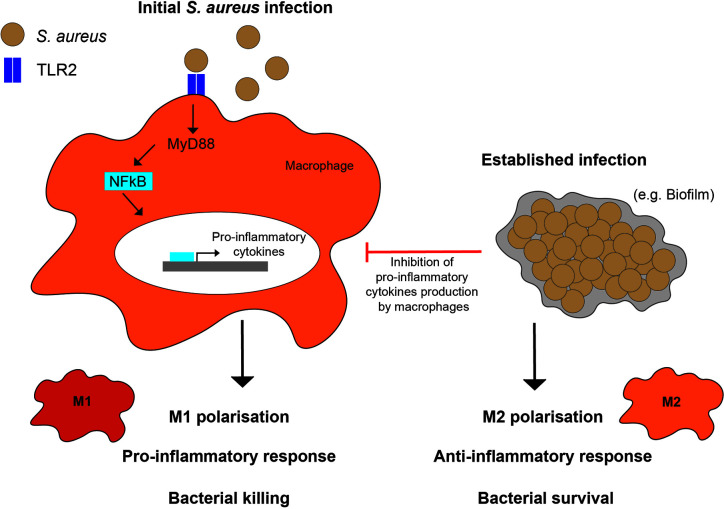
Macrophage polarization responses to *S. aureus*. M1 polarization in response to planktonic (or initial) infections, occurs through TLR2, MyD88, and NF- κB signaling resulting in a pro-inflammatory phenotype and cytokine production. In comparison, M2 polarization in response to established infections, such as biofilms, occurs through inhibition of macrophage pro-inflammatory cytokine production.

Macrophage interactions with *S. aureus* are dependent on the type of pro- or anti- inflammatory immune response elicited (**Figure 3**). Macrophages actively phagocytose planktonic (single bacterial cells) *S. aureus*, but are less able to phagocytose biofilm-associated bacteria (133). This has been extended to keratinocytes, where *S. aureus* biofilms elicit a lesser inflammatory response than planktonic bacteria (134). Furthermore, adequate abscess formation in response to *S. aureus* dermal mouse infection requires M1 macrophages, whereas the presence of M2 macrophages was associated with uncontrolled bacterial spread (135). Changes in macrophage polarization are due, in part, to variations in macrophage stimulation in different *S. aureus* infection scenarios. M1 or M2 polarization leads macrophages to respond to *S. aureus* differently, promoting pro- or anti- inflammatory responses, respectively (136).

### Pro-inflammatory Macrophage Polarization

In some *S. aureus* infections, a robust pro-inflammatory macrophage response can lead to efficient phagocytosis of *S. aureus* after sensing bacterial components. After initial *S. aureus* infection, AMs undergo M1 polarization and secrete pro-inflammatory cytokines (137). M1 macrophages phagocytose and kill intracellular pathogens, generate reactive oxygen species (ROS), nitric oxide (NO) and pro-inflammatory cytokines and can express class II major histocompatibility complex molecules (MHC-II) (138). Macrophages are also capable of longer-term memory in response to recurrent *S. aureus* infection. In localized skin infections, prior infection reduced subsequent infection severity by priming macrophages toward pro-inflammatory phenotypes (139).

Toll-like receptors (TLRs) recognize bacterial components, signaling through MYD88 innate immune signal transduction adaptor and nuclear factor kappa-light-chain-enhancer of activated B cells (NF-κB) to upregulate inflammatory gene expression, including pro-inflammatory cytokines such as TNF-α, IL-6, and IL-1β. TLR2 is particularly important in *S. aureus* infections. In peritoneal macrophages, TLR2 recognizes *S. aureus* PGN, leading to both MYD88 and NF-κB signaling (140). In addition, TLR2 to detects *S. aureus* lipoproteins, shown in keratinocytes where it induces NF- κB activity, lipoprotein activation of TLR2 which was similarly observed in J774 macrophages, leading to pro-inflammatory cytokine production (141, 142). Loss of MYD88 from macrophages inhibited production of TNF-α after exposure to *S. aureus* cell wall (143). Similarly, loss of TLR2 led to reduced pro-inflammatory cytokine expression in peritoneal macrophages infected with *S. aureus* (144). Mice deficient in TLR2 or MYD88 have an increased susceptibility to *S. aureus* infection, as well as a reduction or loss of macrophage expression of pro-inflammatory cytokines TNF-α and IL-6 (140, 145). TLR2 can also be recruited to *S. aureus*-containing phagosomes in macrophages, initiating cytokine production following bacterial degradation (146). CD14 is a co-receptor for TLR2 and, together, they act to promote a pro-inflammatory response, including by M1 polarization of macrophages (147–149). *S. aureus* PGN and LTA bind to CD14 and cause TLR2-mediated activation of NF-κB in HEK cells (150). Studies on *S. aureus* and TLR2 signaling have mainly focused on leukocytes, but *S. aureus* may promote alternate inflammatory responses in other cell types. For example, LTA stimulation of TLR2 on endothelial cells may promote an anti-inflammatory response (151).

As with other aspects of the immune system, TLR2 and NF-κB signaling may be undermined in *S. aureus* infection. Activity of c-Jun N-terminal kinase (JNK) has been associated with TLR2 in *S. aureus* infection, with JNK mediating cell responses to stress. TLR2 signaling through the JNK pathway may be required for macrophage phagocytosis of *S. aureus* (152), however, TLR2-activated JNK signaling in response to *S. aureus* reduces macrophage superoxide generation and enables prolonged survival within the phagosome (153). Similarly, loss of TLR2 in infected peritoneal macrophages was associated with reduced *S. aureus* catalase and superoxide dismutase activity (144). *S. aureus* strains lacking lipoproteins can escape immune recognition by TLR2 (154). Additionally, NF-κB activation is required for macrophage phagocytosis of *S. aureus*, since inhibition of NF-κB blocks bacterial uptake (155). NF-κB activation is reduced in *S. aureus*-stimulated macrophages by activation of a macrophage receptor involved in phagocytosis of apoptotic cells (MerTK), which leads to a reduced inflammatory response to staphylococcal LTA (156). Overall, these studies indicate that pro-inflammatory mediators TLR2 and NF-κB are important in the macrophage response to *S. aureus* and are a target of subversion.

*S. aureus* has further strategies to manipulate macrophage polarization to limit pro-inflammatory responses. Protein kinase B (Akt1) signaling induced by *S. aureus* was shown to decrease macrophage M1 polarization, with mice deficient in Akt1 having improved bacterial clearance. Akt1-deficient macrophages have increased pro-inflammatory cytokine expression and NF-κB activity (157). *S. aureus* induction of macrophage polarization is also modulated by microRNAs. MicroRNA-155 is involved in Akt1-mediated macrophage polarization (157), while microRNA-24, a regulator of macrophage polarization, has reduced expression during *S. aureus* infection (158).

### Anti-inflammatory Macrophage Polarization

In certain *S. aureus* infections, for example in established biofilm infections, an anti-inflammatory response occurs, promoting continued bacterial survival within the host. M2 polarized macrophages and reduced phagocytosis are found in chronic rhinosinusitis, a condition associated with *S. aureus* colonization (159). In mice, *S. aureus* biofilms prevent phagocytosis by macrophages, as well as reduce inflammation through attenuation of pro-inflammatory host responses, favoring an M2 macrophage phenotype (133). In a rat *S. aureus* biofilm periprosthetic joint infection model, an increase in the number of M2 macrophages is observed (160). Additionally, AMs are more likely to become an M2 phenotype in *S. aureus* infections at later time-points in infection (137). Together these reports suggest that established *S. aureus* infections promote M2 polarization.

Antibodies may facilitate *S. aureus*-mediated M2 polarization in chronic rhinosinusitis, whereby bacterial virulence factors cause an increased production of IgE, which in turn promotes M2 polarization (159, 161). Furthermore, biofilm secretion of cyclic di-AMP promotes anti-inflammatory cytokine release from macrophages (162), and *S. aureus* virulence factor secretion from biofilms reduces macrophage phagocytosis (163). *S. aureus* expresses clumping factor A (ClfA) to reduce phagocytosis and subsequent pro-inflammatory response, and this is suggested to be due to immuno-modulation (164).

TLR2, MYD88 and NF-κB signaling are also involved in *S. aureus* biofilm infections and are targeted by *S. aureus* to manipulate the macrophage response. In early control of cranial biofilm infection spread, TLR2 is associated with macrophage IL-1β pro-inflammatory cytokine production, but this signaling was insufficient to clear infection (165), perhaps due to established infection manipulation of the macrophage response. Interestingly, addition of IL1-β to led to increased bacterial growth of biofilm, but not planktonic, *S. aureus*, suggesting that biofilms react to host cytokines to promote survival (166). Catheter-associated biofilm infections in MYD88-deficient mice have increased bacterial burden and dissemination, reduced expression of pro-inflammatory cytokines, and an increased number of M2 macrophages (167). This knowledge has led to production of biofilm treatments which promote a M1, rather than M2 macrophage polarization. Addition of M1 macrophages to the site of an *in vivo* biofilm, led to reduced bacterial burden (168). Remarkably, a therapeutic approach which promotes pro-inflammatory monocyte polarization lead to clearance of established biofilms in mice (169).

### Cytokines in Macrophage Polarization

Macrophages are able to sense cytokines released in the local environment, including cytokines released by nearby activated macrophages in response to *S. aureus* infection. Following binding of cytokines to receptors, the action of the Janus kinase (JAK) and signal transducers and activators of transcription (STAT) signaling pathway mediate transcriptional changes (170). The JAK/STAT pathway is important for activation of macrophages, induction of inflammatory responses, and inhibition of apoptosis.

Exposure of MDMs to *S. aureus* alters the expression of 624 genes, with JAK/STAT signaling changed in early infection (171). JAK/STAT signaling is induced by PGN, leading to phagosome maturation in macrophages containing *S. aureus* (172). Interestingly, in murine influenza and MRSA co-infection, STAT2 is important in macrophage polarization, where STAT2-deficient mice had improved bacterial burden, potentially caused by an increased number of M1 macrophages (173). Human MDMs with an established *S. aureus* infection harbored viable intracellular bacteria within vesicles, however, MDM apoptosis or necrosis was not observed until *S. aureus* escaped to the cytosol (18). Further to this, addition of isolated *S. aureus* PGN can increase anti-apoptotic signals in infected macrophages, likely through the JAK/STAT and NF-κB signaling pathways (174). Macrophages which have phagocytosed *S. aureus* have increased expression of anti-apoptotic genes, enabling continued intracellular bacterial survival (175). To induce this, *S. aureus* upregulates macrophage myeloid cell leukemia-1 (MCL-1) expression, an anti-apoptotic gene which enhances anti-inflammatory cytokine release (176). In contrast to these macrophage studies, the presence of *S. aureus* increases apoptosis in neutrophils (177). Therefore, macrophages may be a prime target for subversion and intracellular persistence.

Interferon-beta (IFN-β) is a cytokine with roles in antimicrobial defense of infected cells, as well as innate and adaptive immunity (178). *S. aureus* can induce a strong IFN-β response in airway infection models, where protein A stimulates IFN-β production, likely *via* TLR9 or NOD2 signaling (179, 180). However, dependent on the *S. aureus* strain used, there is diversity in the IFN response induced (181). Following other routes of infection, *S. aureus* induces variable IFN-β production by macrophages, though IFN-β production or treatment has been shown to be beneficial for the host during *S. aureus* infection. *S. aureus* resistance to macrophage degradation causes the reduced IFN-β production, which is lower than that induced by comparable pathogens (182). This suggests a lack of sufficient IFN-β induction is detrimental to the host. IFN-β production by macrophages is inhibited by TLR2 signaling during *S. aureus* infection. TLR8, an intracellular TLR, senses *S. aureus* RNA in infected macrophages and monocytes, leading to IFN-β production *via* MYD88 signaling (183). TLR2 is a key sensor of *S. aureus* and therefore the antagonistic role of TLR2 and TLR8 signaling may ultimately reduce macrophage IFN-β production.

IL-1β is another pro-inflammatory cytokine with important roles in controlling *S. aureus* infection. In a brain abscess *S. aureus* infection, mice deficient in IL-1β (or TNF-α) were subject to significantly enhanced mortality and greater bacterial burden when compared to wild-type mice (82). In a sub-cutaneous model, mice deficient in MYD88 or IL-1R had significantly bigger lesions and bacterial burden, with IL-1R activation required for neutrophil recruitment to *S. aureus*-infected sites (184). Similarly, mice deficient in IL-1β had larger lesion size, greater colony forming units (CFUs) and reduced neutrophil attraction following *in vivo* cutaneous challenge (185). Supplementation of IL-1β KO mice with recombinant IL-1β restored the mice’s ability to control infection and clear *S. aureus* (185). In contrast, in murine airway *S. aureus* infection, IL1-β is associated with immunopathology (186), and addition of recombinant IL- β reduced bacterial clearance (187). Interestingly, activated platelets which release IL-1β act to enhance macrophage phagocytosis and killing of *S. aureus*, suggesting both platelets and IL1-β have an important role in the phagocyte response (188).

## Mechanisms Used by Macrophages to Kill *S. aureus*

**Figure 4:**
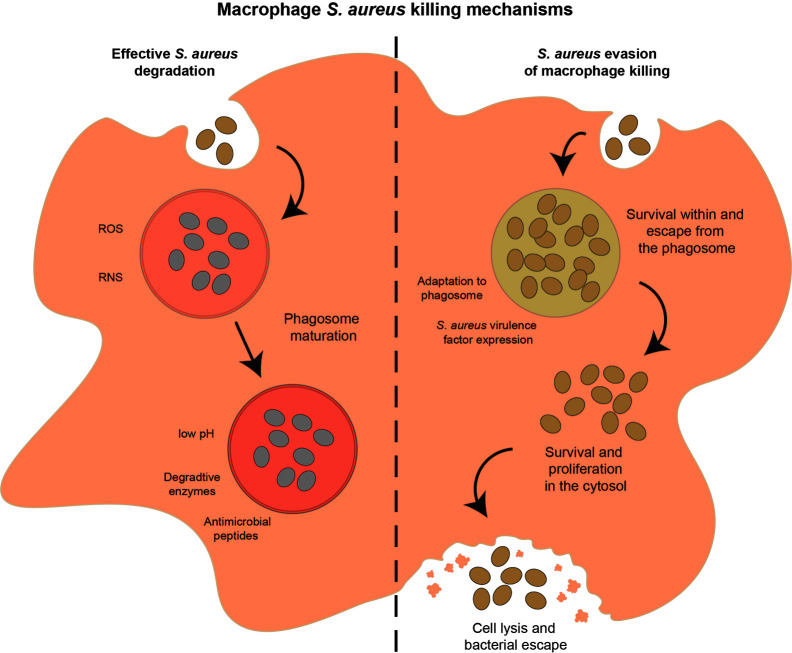
Potential outcomes of the interaction between macrophages and *S. aureus*. After phagocytosis, macrophages can successfully control and degrade *S. aureus* (left hand side of figure) using a range of mechanisms, including ROS and RNS soon after phagocytosis, phagosome acidification, nutrient restriction, release of degradative enzymes and AMPs as the phagosome matures. Alternatively, *S. aureus* can evade macrophage killing mechanisms (right hand side of figure) by adapting to the phagosome environment, expressing a range of virulence factors, or escape from the phagosome and survival in the cytosol, leading eventually to macrophage cell lysis and *S. aureus* dissemination.

Once macrophages are activated, have located and phagocytosed *S. aureus*, the macrophage’s powerful degradative processes are used to kill the bacteria. Macrophages have a range of mechanisms to destroy phagocytosed pathogens (**Figure 4**), including release of reactive oxygen species (ROS), reactive nitrogen species (RNS), enzymes and antimicrobial peptides, as well as acidification of the phagolysosome, nutrient restriction, and autophagy. In addition, macrophages can target extracellular bacteria with extracellular traps.

### Macrophage Production of ROS and RNS

**Figure 5:**
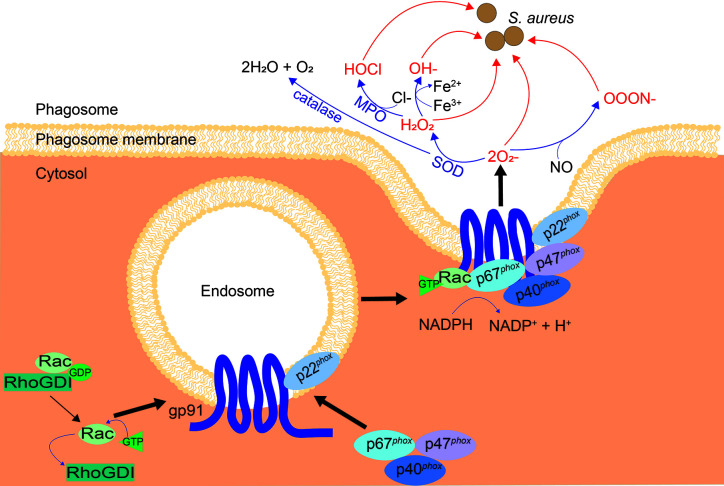
Assembly of NOX2 and subsequent ROS cascade. When inactive, NOX2 components gp91^*phox*^ and p22^*phox*^ are located on vesicles, while inactive Rac and p67^*phox*^, p47^*phox*^ and p40^*phox*^ exist in the cytosol. Upon activation, the cytosolic subunits are localized to phagocytic cups on the endosome membrane, to bind to gp91 and p22^*phox*^. Inhibition of Rac by RhoGDI is reversed, allowing GTP binding and recruitment of Rac to the NOX2 complex. The NOX2 vesicle merges with the membrane of the phagosome and produces superoxide. Superoxide is converted to other ROS: H_2_O_2_ by superoxide dismutase (SOD) and OOON^−^ by interaction with nitric oxide (NO). H_2_O_2_ is converted into HOCl by myeloperoxidase (MPO), OH^−^ by the Fenton reaction, and H_2_O + O_2_ by catalase, as shown.

NADPH oxidase (NOX2) is an enzyme located on the phagosome membrane, assembly of the oxidase is induced which then allows it to catalyze superoxide production $\left({\text{O}}_{2}^{-}\right)$ and subsequent ROS, termed the oxidative burst. Superoxide can be converted into a variety of different ROS (see **Figure 5**), all of which are toxic to some degree. ROS production is considered the key killing mechanism for both macrophages and neutrophils (189), and is important for clearance of *S. aureus* (61, 190).

NOX2 is activated by signals from phagocytic receptors, such as FcγR and macrophage-1 antigen (Mac-1) (22, 191), resulting in electron transfer from reduced NADPH in the cytosol to phagosomal oxygen. Ras-related C3 botulinum toxin substrate (Rac), a small GTPase, is necessary for best operation of NOX2 (192, 193). Rho GDP-dissociation inhibitor (RhoGDI) inhibits Rac, stabilizing the active, GDP-bound form until inhibition is reversed upon NOX2 activation (194, 195). NOX2 has 5 main components (see **Figure 5**), two of which are membrane-spanning: gp91*^phox^* and p22*^phox^*, while three are cytosolic: p40*^phox^*, p47*^phox^*, and p67*^phox^* (196). Gp91*^phox^* and p22*^phox^* are located on Rab11-positive recycling endosomes, Rab5-positive early endosomes and the plasma membrane (197). When NOX2 is activated, the three cytosolic components are recruited to bind gp91*^phox^* and p22*^phox^* at the vesicle membrane *via* phagocytic cups, Rac recruits GTP and binds to p67*^phox^*, and the NOX2 machinery fuses with the nascent phagosomal membrane, producing superoxide. Superoxide production occurs almost immediately, even before phagosomes are sealed, implying that the NOX2 assembly is fast (198, 199).

Although superoxide $\left({\text{O}}_{2}^{-}\right)$ itself is able to destroy bacteria, it is extremely volatile and degrades into hydrogen peroxide (H_2_O_2_), or interacts with nitric oxide (NO) to produce peroxynitrite (ONOO^−^) (189, 200, 201) (**Figure 5**). When iron or other catalytic metals are present in the phagolysosome, possibly due to release from phagosomal proteins, H_2_O_2_ and ${\text{O}}_{2}^{-}$ can react to form a hydroxyl radical (OH^−^); a process known as the Fenton reaction (202–204). Myeloperoxidase (MPO) is the enzyme that catalyzes hypochlorous acid (HOCl) formation from H_2_O_2_ and chloride. This is abundant in neutrophils, although other phagocytes including macrophages express it (201, 205). Hypochlorous acid is thought to contribute to microbicidal activity induced by H_2_O_2_, however, hypochlorous acid is not critical for antimicrobial activity. This is demonstrated by the fact that patients deficient in MPO have similar susceptibilities to bacterial infections as healthy individuals (61, 206, 207). Chronic granulomatous disease (CGD) patients have mutations in one of the subunits of NOX2, resulting in an inability to make ROS. CGD patients are significantly more susceptible to *S. aureus* infection (208). CGD is most commonly due to defects in the genes for gp91*^phox^* or p47*^phox^*, with only 5% of CGD cases due to mutations in genes coding for p22*^phox^*, p40*^phox^* and p67*^phox^* (208–214). Macrophages are implicated in CGD bacterial diseases, since a characteristic of CGD is hepatic abscesses, suggesting the importance of Kupffer cells in control of microbes (215).

In addition to ROS, phagocytes produce RNS. Production of NO radicals is catalyzed by inducible nitric oxide synthase (iNOS) (216). iNOS is only expressed in response to inflammatory stimuli, with IFN-γ being the key cytokine required for iNOS induction in macrophages (216, 217). Upon reaction of NO with superoxide, peroxynitrite is formed, which is toxic to phagocytosed microbes’ proteins and DNA (218, 219). However, mice deficient in iNOS do not suffer a significant increase in intracellular *S. aureus* upon infection, while mice with NOX2 deleted (*cybb* ^−/−^) had significantly increased intracellular burden and hence greater mortality (61, 220, 221). This underlines the importance of NOX2 in defense against *S. aureus*.

There is limited data on the concentration of ROS within the phagosome, much of it relating to neutrophils. Macrophage oxidative burst peaks approximately 30 min post-phagocytosis, although it is maintained for over 60 min (222, 223). In the neutrophil phagosome, the concentrations of ${\text{O}}_{2}^{-}$ and H_2_O_2_ are estimated to be 25 and 2 µM, respectively. However, in the absence of MPO, these concentrations are higher: over 100 µM and 30 µM, respectively (224). This is important, due to macrophages possessing lower concentrations of MPO than neutrophils (205). Macrophages have been estimated to produce 50 µM of ${\text{O}}_{2}^{-}$ and 1 to 4 µM H_2_O_2_ at neutral pH (225). These concentrations were determined using computer modeling to approximate the speed NOX2 can produce ${\text{O}}_{2}^{-}$, the volume of the phagosome, the rate of spontaneous dismutation into H_2_O_2_, and the frequency of H_2_O_2_ diffusion across the phagosome membrane into the cytoplasm (224, 225). There are margins for error at each stage of these calculations, particularly as this assumes homogeneity within the phagosome. *In vitro* measurement of macrophage ROS may be more accurate. However, as these concentrations of ROS are very small and the oxidative burst occurs rapidly, there are difficulties in accurately measuring this.

ROS are also produced by the mitochondria. Mitochondrial ROS (mROS) are, in most cases, the by-product of oxidative phosphorylation. However, more recent studies have demonstrated mROS act as a microbial defense mechanism within macrophages (226, 227). When macrophages were treated with histone deacetylase inhibitors (to test possible downregulation of host immune responses) alongside infection with either *Salmonella* or *E. coli*, intracellular bacterial clearance was enhanced *via* upregulation of mitochondrial ROS, an effect which was reversible upon inhibition of mitochondrial function (226). Furthermore, signaling through Toll-like receptors, specifically TLR1, TLR2, and TLR4, in macrophages leads to recruitment of mitochondria to the phagosome and an alteration in mROS (227, 228). Additionally, when mitochondria were induced to express catalase, *Salmonella* clearance was decreased (227). Likewise, infection of macrophages with *S. aureus* triggered production of mROS, primarily H_2_O_2_, which was delivered to the bacterial-containing phagosome by mitochondria-derived vesicles, contributing to bacterial killing (228). This was determined to be induced by endoplasmic reticulum stress, dependent on TLR signaling and mitochondrial superoxide dismutase 2 (228). Moreover, mitochondria associate to the membrane of *S. aureus*-containing macrophage phagosomes to increase mROS production and activate caspase-1, leading to acidification of the phagosome. However, expression of alpha-hemolysin by *S. aureus* was able to counteract these effects (229). Furthermore, *S. aureus* counteracts recruitment of mitochondria to the macrophage phagosome membrane in a caspase-11–dependent manner, with caspase-11 deletion in mice enabling mitochondrial association with *S. aureus* vacuoles, increased mROS and improved bacterial clearance (230).

### *S. aureus* Response to ROS

ROS can damage biomolecules including essential enzymes and DNA (225). However, bacteria have evolved mechanisms to withstand ROS and RNS. Staphylococcal peroxidase inhibitor (SPIN) is secreted by *S. aureus*. SPIN attaches to and incapacitates human MPO (231). Structural analysis revealed that SPIN acts as a “molecular plug”, occupying the active site of MPO and thus refusing entry to the H_2_O_2_ substrate (231). Expression of SPIN was maximal within a phagosome, which is the location of MPO, and *S. aureus* mutants deficient in SPIN have reduced survival following phagocytosis when compared to wild-type *S. aureus* (231), this was demonstrated with neutrophils but is likely to occur in macrophages.

*S. aureus* possesses two superoxide dismutases which incapacitate superoxide radicals, superoxide dismutase A (SodA) and superoxide dismutase M (SodM) (232). Some studies have identified SodA and SodM as important for *S. aureus* virulence (232, 233), while others show only marginal effects (234, 235). Manganese ions act as a co-factor for SodA and SodM, upregulating superoxide dismutase activity without affecting transcription, and *S. aureus* is more susceptible to manganese starvation in the absence of these proteins (232, 236). SodA is valuable in resisting superoxide stress in the presence of manganese, while SodM is crucial when in manganese-scarce environments (236). SodM is not present in other staphylococci, and this role of inhibiting host ROS during manganese restriction may explain why *S. aureus* has acquired a second superoxide dismutase.

Resistance to oxidative stress in *S. aureus* is mediated, in part, by transcriptional regulators. Peroxide regulator (PerR) is an important regulator which controls a regulon of many antioxidant genes. In particular, alkylhydroperoxide reductase (AhpC) and catalase (KatA) are involved in resisting peroxides and H_2_O_2_ respectively (237). The genes encoding these two proteins are regulated in a compensatory manner: mutation in *ahpC* enhanced (rather than reduced) H_2_O_2_ resistance, as *katA* is upregulated by removal of PerR repression (237). AhpC was similarly able to compensate for *katA* mutation. Deletion of both *katA* and *ahpC* caused a significant growth defect, with *S. aureus* unable to remove intra- or extracellular H_2_O_2_, meaning H_2_O_2_ accumulated to toxic levels in the media (237). *S. aureus* mutants lacking two component regulator staphylococcal respiratory response AB (SrrAB*)* were more susceptible to H_2_O_2_, with *katA* and *ahpC* transcriptionally downregulated (238). Susceptibility to H_2_O_2_ was reversed by iron sequestration or *perR* repressor gene deletion (238). Another study showed that the *msaABCR* operon of *S. aureus* regulates expression of genes involved in oxidative stress (239). Staphyloxanthin, a carotenoid pigment, is a strong antioxidant which is regulated by cold shock protein (CspA), alongside the organic hydroperoxide resistance gene which defends specifically against oxidative stress caused by organic hydroperoxides. This implies involvement of ROS resistance genes in persistence of *S. aureus* (239).

A transposon screen found there were five *S. aureus* regulons which are crucial for NO resistance (240). Flavohemoglobin (Hmp) is necessary for resistance to NO in some bacterial species, because it acts as a denitrosylase, removing NO (241). This is strictly controlled, as Hmp expression in the absence of NO leads to enhanced oxidative stress (242). Nitrite-sensitive repressor (NsrR), is the NO-sensing transcriptional regulator of Hmp used by many bacteria to detect and react to NO (242–245). *S. aureus* does not possess NsrR, instead, the two-component regulator SrrAB controls Hmp (243). Additionally, modifications to *S. aureus* metabolism may increase bacterial NO resistance. Infection of RAW 264.7 cells with a *S. aureus* TCA cycle mutant had reduced NO production and iNOS activity when compared to wild-type *S. aureus* (246).

*S. aureus* can take advantage of host signaling in order to escape oxidative killing. In wild-type mice, *S. aureus* phagocytosis by macrophages led to JNK activation in a TLR2-dependent manner; JNK activation caused inhibition in superoxide production, impairing the ROS cascade and prolonging survival of the bacteria. When TLR2-deficient mice were used, the macrophages were more able to readily kill *S. aureus* (153). TLR2 expression is higher in *S. aureus*-infected macrophages (144), and *S. aureus* were more able to escape killing by peritoneal macrophages when anti-TLR2 antibodies were used (247).

*S. aureus* produces lipoic acid, which also restricts ROS and RNS production by macrophages, to enhance bacterial survival (248). Lipoic acid is a metabolic cofactor which is synthesized by the lipoic acid synthetase (LipA), which limits macrophage activation by reducing TLR1 and TLR2 activation by bacterial products (249). A *S. aureus lipA* deletion mutant caused significantly more TLR2-dependent pro-inflammatory cytokine production (249). Exogenous lipoic acid can reduce neutrophil oxidative burst through radical binding as well as recycling antioxidants, inhibiting NF-κB transport into the nucleus, and reducing production of inflammatory cytokines (250–254). Macrophages which were recruited to the site of infection with the *lipA* mutant produced significantly greater amounts of ROS and RNS than those attracted to sites infected with wild-type *S. aureus* (248); in this case, ROS and RNS (but not mitochondrial ROS) were important for controlling *S. aureus lipA* infection (248). This suggests that lipoic acid production by *S. aureus* promotes persistence of the bacteria.

### Macrophage Phagosomal Acidification

Acidification of the phagosome is another key mechanism involved in killing phagocytosed bacteria. A low phagosomal pH may directly affect *S. aureus* survival, since bacterial growth is reduced at pH 4.5 (255). Additionally, acidification has an important impact on phagosomal enzymes, for example cathepsins, which have optimal efficacy at low pH. Phagosomal enzymes are discussed in detail in the enzymes section below.

Macrophage phagosome acidification is generated by an influx of protons (H+) into the phagosome by vacuolar-type proton transporting ATPase (v-ATPase), which is present in phagosome membranes (256). The action of v-ATPase reduces the pH of endosomes and lysosomes to ~6 and ~4.5, respectively (257). Fusion of endosomes and lysosomes, which are enriched with v-ATPase, is an important part of phagosome maturation, the continued delivery of v-ATPase causes increasing acidification throughout sequential stages of phagosome maturation (258). In addition to this, the permeability of the phagosome to protons is important in maintenance of low pH, therefore as phagosomes mature, proton permeability is decreased to preserve acidification (259). However, phagosome acidification commences before lysosomal fusion events occur, demonstrating that v-ATPase is also present at an earlier stage is phagosome maturation (256). Indeed, v-ATPase is found on the plasma membrane of phagocytes where it is used to maintain cytosolic pH (260, 261). The v-ATPase present in plasma membranes are likely internalized during phagocytosis and responsible for acidification at very early timepoints of phagocytosis, with additional v-ATPase delivered during phagosomal maturation leading to increased acidification.

Phagosomal acidification is well documented in *S. aureus* infection. In *S. aureus*-infected murine peritoneal macrophages, the phagosomal pH is reduced to 5.7 to 6 within 6 to 8 min of infection, and this is dependent on v-ATPase (256, 259). Indeed, the average phagolysosomal pH of RAW 264.7 cells infected with *S. aureus* was measured as 5.43 12 hours post-infection (262). *S. aureus* phagocytosed by Kupffer cells is trafficked to an acidified phagosome, as demonstrated in intravital imaging of murine infections (263). Another study shows that *S. aureus* peptidoglycan can induce macrophage phagosome maturation through JAK-STAT signaling (172). Low pH is also important in efficiently killing *S. aureus* in neutrophils (264). Non-professional phagocytes, including epithelial cells and endothelial cells, are also shown to traffic *S. aureus* to an acidic phagosome (19, 20, 265, 266). Phagosome maturation proteins are involved in *S. aureus* degradation. For example, copper metabolism gene MURR1 domain (COMMD) proteins regulate both intracellular trafficking and transcription factors. Kupffer cells effectively kill *S. aureus*, where phagosomes mature in a COMMD10-dependent manner, required for phagosome acidification and optimal bacterial killing (267).

*S. aureus* can adapt to the acidic phagosome, with recent studies suggesting that exposure to acidification may even promote intracellular bacterial survival. *S. aureus* can survive and replicate within mature acidic phagosomes, as demonstrated using murine macrophages and human MDMs (16). *S. aureus* can survive and replicate within murine AMs, and inhibiting phagosome acidification caused a small drop in bacterial survival (268). Similarly, THP-1 cells were also used to show that inhibiting phagosome acidification reduced *S. aureus* survival, where exposure to low pH was shown to induce virulence factor expression (269). THP-1 cells which are deficient in phagosomal acidification had improved bacterial killing of *S. aureus* strain USA300, although not the Newman strain (270). Phagosomal acidification has even been proposed to be requisite for *S. aureus* intracellular survival, the bacterial GraXRS regulatory system is used to sense low pH, where *S. aureus* promotes adaptive responses enabling bacterial growth within the phagosome, shown to be required for bacterial survival within murine Kupffer cells *in vivo* (262).

Other studies show that macrophages with phagosomes containing *S. aureus* do not acidify appropriately. Reduced acidification of the phagosome was observed in THP-1 cells when infected with *S. aureus*, in comparison to *E. coli* or *S. pneumoniae*, and the authors suggest that reduced acidification may precede bacterial escape (73). *S. aureus* has also been shown to reside within non-acidified vesicles in epithelial cells (271). Presence of other material in the phagosome with *S. aureus* reduced the acidification of Kupffer cell phagosomes, promoting *S. aureus* survival (272). Whether phagosomes containing *S. aureus* properly acidify, leading to beneficial or detrimental effects on the host, likely depends on multiple factors; cell types, bacterial strains, timepoints and phagosomal markers studied, as well as the antagonistic roles of ROS production and proton influx discussed below.

There is evidence that the actions of phagosomal NOX2 and v-ATPase are antagonistic. In the early stages of phagosomal maturation, ROS production by NOX2 may buffer acidification through rapid consumption of protons. The oxidative burst is therefore intrinsically linked to phagolysosome acidification (73, 273), and, as such, oxidation can delay phagosomal maturation (223). In neutrophils, NOX2-dependent reduction of phagosome acidification is caused by proton consumption, as well as decreased v-ATPase recruitment to the phagosome and increased membrane permeability to protons (274). Caspase-1 limits the antagonism of NOX2 and v-ATPase in macrophages infected with *S. aureus* by regulating NOX2 activity (through cleavage of NOX2 components) to promote phagosomal acidification (275). Interestingly, phagosomes of pro-inflammatory M1-like human macrophages acidify less in comparison to anti-inflammatory M2-like macrophages, due to sustained NOX2 retention on the phagosome and associated proton consumption by the ROS produced (223). Since proteolytic enzymes are less functional at higher pH, the antagonistic effects of NOX2 activity on pH may reduce the degradative capacity of the phagosome. It has been hypothesized this ensures ROS-mediated destruction of microbes before subsequent degradation of microbial products (22). Antigen presenting cells present antigens to the adaptive immune system. In macrophages and dendritic cells, increased NOX2 activity is associated with reduced proteolysis (273, 276–278), meaning antigens are retained longer for improved presentation to adaptive immune cells (279). There appear to be multiple mechanisms causing NOX2 and v-ATPase antagonism, which differ between cell types, likely due to their different roles. As limited studies use macrophages, which have important roles in antigen presentation, there remain many unanswered questions.

### The Role of Macrophage Enzymes in Controlling *S. aureus* Infection

Mature phagosomes may contain hydrolytic enzymes that kill bacteria efficiently. These include proteases, lipases, phosphatases and glycosidases. These enzymes have optimal efficacy in acidic conditions (280, 281). The acidification of mature phagosomes is discussed above.

The phagosome of macrophages can contain lysozyme, which is an enzyme that cleaves bacterial peptidoglycan. *S. aureus* is resistant to lysozyme due to acetylation of PGN by O-acetyltransferase (OatA) (282). PGN acetylation may also reduce activation of the NLRP3 inflammasome, avoiding induction of IL-1β (283). IL-1β is produced by phagocytes in response to inflammasome activation and is a key weapon in the arsenal of the immune system against *S. aureus* (82). The NLRP3 inflammasome is activated by exposure to phagocytosed PGN (283). In order to trigger this response, PGN must be partially digested by lysozyme. Thus, the ability of OatA to induce resistance to lysozyme suppresses activation of the NLRP3 inflammasome and subsequent IL-1β induction, demonstrated both *in vitro* and *in vivo* (282, 283). Underlining the importance of the inflammasome, mice deficient in inflammasome component apoptosis-associated speck-like protein containing a caspase recruitment domain (ASC), failed to induce IL-1β expression and suffered increased lesion size, increased CFUs and decreased neutrophil attraction upon challenge with *S. aureus* (185). Interestingly, the route of *S. aureus* infection influences the role of the inflammasome. *S. aureus* is known to commandeer the NLRP3 inflammasome during lung infection to aggravate pathology (284), while inflammasome activation during skin and soft tissue infection leads to clearance of the bacteria (285). For further discussion of inflammasome involvement in *S. aureus* infection, see (286).

Cathepsins are proteases found in the lysosomal compartment which are highly expressed in macrophages. Cathepsin D-deficient mice were more susceptible to infection with intracellular pathogen *Listeria monocytogenes*, which survived phagosomal killing significantly more than in wild-type mice (287). Cathepsin D is thought to act by degrading secreted bacterial virulence factors (287). Cathepsin G secreted from neutrophils damages *S. aureus* biofilms (288). Cathepsins have been shown to be involved in macrophage *S. aureus* engulfment and killing, with cathepsin L indicated as an inducer of non-oxidative killing, and cathepsin K important in induction of IL-6 production (289). The method of cathepsin-mediated *S. aureus* killing is thought to be direct proteolytic damage (289). In addition to modulating IL-6 production from macrophages, cathepsins can also influence IL-1β production (290, 291). This has been demonstrated in a bone marrow-derived macrophage model of *Mycobacterium tuberculosis* infection, whereby cathepsin release was critical for inflammasome activation and IL-1β production (292), providing evidence that this may be a common mechanism to control intracellular bacteria.

A further example of macrophage antimicrobial enzymes is phospholipases, which influence immunomodulatory compounds and attack the membrane of microbes. For example, the group IIA secreted phospholipase A2 (IIA-sPLA2) has strong antimicrobial activity against bacteria, especially Gram-positives (293, 294). IIA-sPLA2 mediates *S. aureus* cell membrane and cell wall damage, leading to bacterial cell death (293). Specifically, IIA-sPLA2 targets phosphatidylglycerol in the bacterial cell membrane, with the strong positive charge of PLA2 binding efficiently to the negative charge of bacteria (295). A *S. aureus* mutant deficient in wall teichoic acid (WTA) was around 100-fold more resistant to IIA-sPLA2 killing, likely caused by reduced access to the cell surface for PLA2 binding (296). Interestingly, one study found that *S. aureus* degradation was only successful when IIA-sPLA2 was accompanied by neutrophil NOX2 activity, independent of MPO (297). Since macrophages, unlike neutrophils, produce IIA-sPLA2, these complementary oxygen-dependent and -independent killing mechanisms may play a role in macrophage-mediated *S. aureus* degradation.

### Antimicrobial Peptides in Macrophage Defense Against *S. aureus*

Antimicrobial peptides (AMP) tend to be positively charged and damage the membrane of pathogens. In order to defend itself against AMPs, *S. aureus* modifies its cell membrane by reducing the negative charge to repel cationic AMPs, thus minimizing electrostatic interactions. Negatively-charged lipids in the cytoplasmic membrane have positively-charged lysine added to them, catalyzed by enzyme multiple peptide resistance factor (MprF), with a similar effect carried out by addition of D-alanine onto cell WTAs, produced by the *dlt* operon gene products (298–300). *S. aureus* which have accumulated extra copies of the *dlt* operon possess teichoic acids with more D-alanine, and hence a greater positive surface charge and lesser susceptibility to binding and damage by cationic AMPs (300). Mutants that are more susceptible to AMPs display teichoic acids that lack D-alanine, when compared to wild-type bacteria (300, 301), meaning they were more attractive to cationic AMPs, including human defensin HNP1-3 (300). Through this mechanism, MprF was found to enable resistance to defensins and protegrins (299). *S. aureus* with an MprF deficiency were significantly attenuated in mice and killed with considerably more efficiency by human neutrophils, as well as displaying an inability to grow within macrophages (262, 299). *S. aureus* has also been shown to counteract the activities of AMPs by integrating lysyl-phosphatidylglycerol in *S. aureus* cell membranes, and expressing the AMP transporter VraFG, which promotes resistance to cationic AMPs (301).

AMP hepcidin is released by macrophages (and neutrophils) *in vitro* and *in vivo* upon microbial detection *via* TLR-4 in order to limit iron availability (302). Furthermore, cytokines including TNF-α, IFN-γ, IL-1, and IL-6 also induce iron modulation (303–309). For example, upon detection of bacteria, IL-6 is stimulated to directly induce expression of hepcidin, leading to hepcidin binding ferroportin, an iron transporter, which causes ferroportin degradation. Degradation of ferroportin reduces the concentration of circulating iron, although it may increase intracellular iron which may have a beneficial effect on intracellular bacteria (310–312). However, other studies show that hepcidin mRNA was induced in RAW 264.7 macrophage-like cells when stimulated with IFN-γ and mycobacteria, but not when the stimulating cytokine was either IL-6 or IL-1β (313).

Calprotectin, an AMP present in monocytes, neutrophils and early macrophages (314, 315), sequesters metal ions to reduce their bioavailability. This has been particularly well-documented for iron, manganese and zinc (316–318). In fact, calprotectin is able to use this sequestration of metal ions to successfully inhibit growth of *S. aureus* in a mouse abscess model (317, 319). The *S. aureus* manganese transporters MntH and MntABC have been shown to work synergistically to overcome manganese scavenging by calprotectin (320). To overcome zinc scavenging, *S. aureus* expresses two zinc transporters and the metallophore staphylopine (321). Furthermore, bone marrow derived macrophages (BMDM) which were primed with calprotectin were induced to produce IL-6, CXCL1 and TNF-α, while BMDMs without calprotectin had a significantly reduced pro-inflammatory response (322).

Cathelicidins have multiple functions, including inducing antimicrobial action, guiding immune cell differentiation toward proinflammatory effects and steering chemotaxis (323). Cathelicidins opsonize bacteria to significantly enhance phagocytosis of *S. aureus* by macrophages *in vitro* by up to 10-fold (324). Cathelicidin fowlcidin-1 induces expression of pro-inflammatory cytokines in order to activate macrophages protecting mice from death in a normally lethal intraperitoneal MRSA infection (325). Furthermore, cathelicidin LL-37 improves macrophage killing of *S. aureus*, with LL-37 endocytosis by macrophages correlated with enhanced ROS production and lysosomal fusion (326).

### Metal Accumulation and Restriction in the Phagosome

Metal ions are essential for bacterial metabolic activity, reproduction and oxidative stress defense (327). However, metal ions are also involved in production of ROS and RNS (328). Immune cells reduce availability of metal ions and alter the metabolic use of metal ions, termed ‘nutritional immunity’ (204, 328). Nutritional immunity studies show limiting availability to metal ions inhibits bacterial growth (329).

The role of metals in phagocytic microbial control has been extensively reviewed (330). Briefly, metal ions iron and manganese are restricted from the phagosome, while copper and zinc are used to overwhelm microbes with toxicity (330).

Although metal ions are essential and contribute to the functionality of many bacterial enzymes, high concentrations can be toxic to bacteria by enabling ROS production, as well as possessing high-affinity to metal-binding portions of proteins which can lead to bacterial enzymes binding excess metal ions, interfering with enzyme function (331). For example, copper has been described to be toxic to microbes by replacing iron ions in essential enzymes, as well as facilitating the production of hydroxyl radicals (332).

Manganese sequestration was found to be crucial for maximal inhibition of *S. aureus* growth *in vitro* (319). Manganese acquisition is essential for *S. aureus* survival (333–335), and is important for oxidative stress resistance due to acting as a cofactor for superoxide dismutase enzymes (334, 336). *S. aureus* with mutations in manganese transporters MntC or MntE were unable to resist methyl viologen (which interacts with electron donors to produce superoxide) likely due to an inability of the superoxide dismutases to function properly in the absence of manganese (334, 335). Similarly, mutations in manganese transporters MntABC and MntH resulted in *S. aureus* with increased sensitivity to methyl viologen, which was reversed by manganese supplementation (337).

Iron is essential for the functioning of many vital bacterial enzymes. However, when present in abundant quantities, iron catalyzes the generation of hydroxyl radicals *via* the Fenton reaction (204). Macrophages control iron homeostasis in part *via* NO-facilitated nuclear factor erythroid 2–related factor 2 transcription factor activation which upregulates the iron exporter ferroportin-1 (338). Macrophages with the gene for iNOS deleted had significantly higher concentrations of iron due to less expression of ferroportin-1, and this iron was able to be harnessed by intracellular *Salmonella* (338). *S. aureus* overcomes iron restriction by production of siderophores, which are able to competitively bind to iron to prevent sequestration by iron-binding host molecules such as lactoferrin and transferrin. In fact, both staphyloferrin A and staphyloferrin B have been shown to displace iron from transferrin (339, 340). Staphylococcal iron-regulated transporter (SirABC) is a transporter of *S. aureus* staphyloferrin B, and has been found to be expressed in response to oxidative and nitrative stress, providing protection from oxidative killing (341). These effects underline the importance of iron in macrophage antimicrobial defense.

Host cytokines are involved in regulating metal ion homeostasis in phagocytes. A number of cytokines identified as particularly important in defense against *S. aureus*, IL-1, IL-6, IL-10, and TNF-α, can act to make iron less available in monocytes and macrophages (82, 328). Unfortunately, this can have the unintended side-effect of anemia in the host. Accumulation of iron was correlated with reduced expression of pro-inflammatory cytokines TNF-α, IL-12, and IFN-γ, leading to an inability to control intracellular bacteria. This effect was reversed upon addition of an iron chelator (338). Host expression of GM-CSF activates the sequestration of zinc, leading to enhancement of H^+^ channels in the phagosome membrane, and induces NOX2 to produce ROS (342). Pro-inflammatory cytokine IFN-γ has been shown to upregulate expression of copper transporter Ctr1. This stimulates copper influx, which was found to be necessary for efficient bactericidal activity (343).

### Nutrients in Control of *S. aureus* Infection

Nutrients, such as fatty acids and amino acids, are important for *S. aureus* survival. Additionally, fatty acids can also be antimicrobial. The host environment can be unfavorable for bacterial growth, as nutrients are restricted. Therefore, bacterial metabolism, essential compound scavenging, and defense against antimicrobial fatty acids is associated with *S. aureus* survival during infection.

Amino acid availability is critical for *S. aureus* growth, in fact many staphylococcal strains isolated from human skin are auxotrophic for multiple amino acids (344). In bovine mastitis infections, at least seven amino acids were required for *S. aureus* growth (345). Following exposure to H_2_O_2_, *S. aureus* amino acid metabolism is altered, likely with increased amino acid consumption promoting bacterial survival (346). Also, amino acid catabolism enables *S. aureus* survival within abscesses, where glucose supply is limited (347). In macrophages, *S. aureus* may induce host cell autophagy to increase metabolite availability to support intracellular proliferation (348). *S. aureus* is able to incorporate exogenous fatty acids into bacterial membranes (349). Host low-density lipoprotein (LDL) can be used as a fatty acid supply by *S. aureus*, removing the need for bacterial synthesis of fatty acids (350). Indeed, *S. aureus* uses host derived fatty acids when available, which is associated with higher levels of staphyloxanthin; thus saving energy in fatty acid synthesis and allowing virulence factor expression (351). Alternatively, *S. aureus* may obtain nutrients from the extracellular milieu *via* macrophage macropinocytosis, inhibition of which reduced *S. aureus* intracellular replication (352). Macrophage micropinocytosis occurs constitutively (353), indicating a potential route of nutrition for intracellular *S. aureus*.

In macrophages, the role of host lipids in infection with intracellular pathogens has been comprehensively reviewed; highlighting how fatty acids and their derivatives can have positive and negative consequences for pathogens, and that lipid metabolism changes with macrophage polarization (354). Antimicrobial fatty acid production by HeLa cells is protective against *S. aureus* infection (355). Leukocytes may also generate bactericidal fatty acids against *S. aureus* biofilms (356). Multiple unsaturated fatty acids are bactericidal against *S. aureus*, including linolenic acid and arachidonic acid, and fatty acid efficacy increases with greater unsaturation (357). Therefore, poly-unsaturated fatty acids (PUFAs) have greater antibacterial properties. Mice fed high levels of PUFAs had increased survival and reduced bacterial burden, along with an improved neutrophil response, following *S. aureus* sepsis infection (358). PUFA bactericidal effects against *S. aureus* were suggested to occur through a mechanism involving ROS (359). Arachidonic acid is a PUFA released at the same time as the oxidative burst in phagocytes, contributing to *S. aureus* killing. Arachidonic acid is oxidized to create electrophiles which are toxic to *S. aureus*, which is likely exasperated by ROS produced during the oxidative burst (360). Fatty acid cis-6-hexadecenoic acid is found on the skin and inhibits *S. aureus* survival, so, *S. aureus* increases defense gene expression (361).

In response to unsaturated fatty acids, *S. aureus* expression of genes involved in membrane stability and metabolism is increased as part of the stress response, indicating that fatty acids disrupt both bacterial lipid membranes and bacterial metabolism (362). *S. aureus* can increase resistance to fatty acids by reducing exogenous fatty acid incorporation into lipid membranes (363). *S. aureus* also uses fatty acid modifying enzyme (FAME) to inactivate fatty acids in abscesses (364). When host fatty acids are incorporated into the membrane of *S. aureus*, expression of the T7SS is increased, leading to virulence factor export (365). Furthermore, *S. aureus* expresses fatty acid resistance genes which confer resistance against linolenic acid and arachidonic acid (366). Studies on the role of macrophage unsaturated fatty acids and PUFAs on *S. aureus* are lacking, perhaps due to focus on the major role of fatty acids on the skin. However, further research may be beneficial due to fatty acid presence in biofilms, as well as fatty acids being associated with the phagocyte oxidative burst.

### Macrophage Autophagy in *S. aureus* Infection

Macroautophagy (autophagy) is the cellular lysosomal self-degradation of damaged or unwanted components; however, autophagy components can be used to target pathogens for degradation. In recent publications, macrophage autophagy machinery has been revealed as an important host target which *S. aureus* is able to manipulate (367, 368). Autophagy proteins may be present at multiple stages of *S. aureus* infection, from phagosomes and autophagosomes, to targeting cytosolic bacteria. Autophagy machinery is more abundant in *S. aureus* infected murine macrophages (369). *S. aureus* can manipulate autophagy to promote survival and subsequent escape from within phagocytic cells in an Agr-dependent manner, potentially enabling persistence in sepsis infections (367). Similarly, high expression of vancomycin resistance-associated sensor/regulator also induces autophagy to a greater extent and is associated with increased intracellular survival in macrophages (368). Therefore, the extent autophagy may benefit *S. aureus* appears to be dependent on the virulence of individual strains. In a murine lung infection model, inhibiting autophagy with drug treatments reduces bacterial burden in the lung (370), again indicating that autophagy is beneficial for *S. aureus*. In agreement, in diabetic settings associated with increased autophagy, a larger number of autophagosomes containing *S. aureus* are observed, and blocking lysosome fusion to autophagosomes promotes *S. aureus* survival in macrophages (371). In bovine macrophages, *S. aureus* infection increased the number of autophagosomes, leading to increased bacterial survival, which also suggests later stages of the autophagy pathway are blocked (372). Together these data suggest that *S. aureus* resides within an autophagic vesicle within macrophages, possibly by blocking autophagy pathway advancement, thereby inhibiting macrophage-mediated killing. The role of autophagy in *S. aureus*-infected neutrophils is less clear, although it seems that the involvement of different autophagic machinery involved at early and late autophagy stages may lead to alternative bacterial outcomes (65, 373). Autophagy also represents a potential therapeutic target in *S. aureus* infection, whereby selenium may promote autophagy within macrophages to an extent that overcomes the bacterial block of the autophagy pathway (374). The involvement of autophagy in macrophage-*S. aureus* interactions is clearly demonstrated, but whether it directly affects infection outcome has yet to be examined in detail and remains an interesting area with possible therapeutic potential.

### Macrophage Apoptosis-Associated Killing Is Deficient in *S. aureus* Infections

Macrophage apoptosis-associated bacterial killing is important for the clearance of a number of pathogens such as *M. tuberculosis* (375) and, in particular, *S. pneumoniae* (376, 377). It is suggested that macrophage phagocytic ability outpacing bactericidal activity leads to permeabilization of the phagolysosome, leading to cathepsin D release, which causes a reduction in anti-apoptotic Mcl-1 expression and, eventually, macrophage apoptosis (378). Interestingly, this mechanism is not observed following phagocytosis of *S. aureus*. Indeed, phagocytosis of *S. aureus* is associated with upregulation of both B cell lymphoma 2 gene (BCL2) and Mcl-1 (175), leading to decreased apoptosis. It has also been proposed that *S. aureus* inhibition of phagolysosome acidification and maturation circumvents apoptosis, enabling persistence in macrophages, although the exact mechanism remains unclear (73). Since persisting intracellular *S. aureus* can be found in the cytoplasm, further studies have suggested that *S. aureus* escapes the phagolysosome, which was associated with increased antiapoptotic host cell proteins (379). This is in stark contrast to extracellular *S. aureus*, which actively promotes macrophage apoptosis by releasing α-hemolysin or Panton-Valentine leucocidin (PVL) toxins (380–382). Detailed discussion of *S. aureus* virulence factors is outside the scope of this review (22, 24, 383, 384).

### Macrophage Extracellular Traps and *S. aureus*

Extracellular traps (ET) are protrusions of chromatin, histone proteins, DNA, proteases and AMPs, that ensnare bacteria and form an important part of the immune response to infection (385), first described in association with neutrophils (386). Neutrophil extracellular traps (NET) vary in their formation (fast or slow) (387, 388) and composition (chromatin or mitochondrial DNA) (389). Original descriptions of NETs demonstrated formation over 3 hours by the destruction of the nuclear membrane leading to death of the neutrophil. More recently, certain NETs were shown to form within 60 min by extrusion of vesicles containing chromatin in a rapid and oxidant independent mechanism (387). Moreover, NETs can be formed of mitochondrial DNA rather than chromatin, in a mechanism that is independent of cell death but was associated with increased survival of neutrophils (389). This allows the neutrophil to continue to contribute to the host immune response. NET formation can be induced in response to a number of different stimuli such as LPS, IL-8, complement factor C5a, and bacteria including *S. aureus* (386, 387, 389).

There is a growing body of evidence that many different innate immune cells are capable of producing ETs to control bacteria, including eosinophils (390), mast cells (391) and macrophages (392). Macrophage ETs (MET) play a role in host defense. Bovine monocyte-derived macrophages form METs in response to *Mannheimiae haemolytica* and to its leukotoxin (LKT) (392). Interestingly LKT did not induce MET formation by bovine alveolar macrophages, suggesting that macrophage differentiation determines the ability to trigger MET formation. Additionally, MET formation was demonstrated by bovine macrophages in response to *Histophilus somni* (393)*. E. coli* also induced MET formation in RAW 264.7 macrophages, which was NADPH oxidase-dependent (392). Similarly, METs were induced in J774 cells in response to *E. coli* and *Candida albicans*, with authors suggesting that the role of METs is to slow dissemination of microbes (394). However, phagocytosis and MET formation have been observed to coincide for control and clearance of *C. albicans* (395).

MET formation can be stimulated by the use of statins. The pre-treatment of human and murine macrophages with statins is associated with increased *S. aureus* killing (396). In neutrophils the proposed mechanism for this enhanced clearance was a significant increase in NET formation, leading to increased *S. aureus* entrapment. A similar result was observed in PMA-stimulated RAW 264.7 macrophages. Initially, NETosis was considered to culminate in cell death, but emerging evidence has shown in neutrophils NETs can be independent of cell death (389). However, the formation of METs appears to trigger a form of cell death as the macrophage exhibits loss of membrane integrity (396, 397), and this may be associated with caspase-1 activity (398), although data on this are sparse. This is further evidence that MET formation may act to slow the dissemination of infection and allow neighboring macrophages to phagocytose bacteria (394).

Pathogens have evolved mechanisms to overcome ETs. For instance, *Streptococcus pneumoniae* evades NETs by producing endonuclease EndA, which degrades the DNA in the NET (399). Similarly, *S. aureus* secretes nuclease and adenosine synthase, leading to the conversion of NETs to deoxyadenosine and in turn triggering caspase-3–mediated cell death to cause non-inflammatory macrophage apoptosis (400–402). This effectively leads to the removal of phagocytic cells from the site of infection allowing abscess formation.

### Extracellular Vesicles From *S. aureus* and Macrophages

Extracellular vesicles (EV) are known to be secreted by a number of different Gram-positive bacteria, including *S. aureus* (403). These bacterial EVs have been shown to contain a variety of virulence factors and provoke significant immune responses. Indeed, one of the first descriptions of *S. aureus* EV demonstrated they could contain β-lactamases, which enabled surrounding bacteria to withstand ampicillin (404). EVs also trigger apoptosis. *S. aureus* EVs deliver virulence factors such as α-hemolysin to macrophages, leading to NLRP3 inflammasome-induced pyroptosis (host cell death) (405), which can be inhibited by fosfomycin (406). In a murine model, EVs were shown to cause atopic dermatitis-like inflammation in the skin (407). EVs secreted by *S. aureus* can also cause mastitis (408). Furthermore, it has been postulated that EVs could be a target for vaccine development (409). The use of statins in a murine survival model decreased macrophage responses to *S. aureus* EVs, suggesting a possible novel therapeutic approach (410).

In addition to extracellular vesicles (EV) secreted by *S. aureus* to subvert the host, there are numerous examples of immune cells releasing EVs. These can vary in size and content and have been isolated from several different cell types, including macrophages. EVs are primarily thought to act as communicating mechanisms allowing an orchestration of immune responses and are an important part of the junction between the adaptive and innate immune responses (411). Indeed, EV from macrophage infected with *M. bovis* modulated T lymphocytes responses (412). When macrophages were infected with *M. tuberculosis*, the content of the EV changed to confer decreased inflammatory cytokine release and decrease lung mycobacterial load (413). Furthermore, during infection with hepatitis C virus, macrophages secreted EVs that inhibited viral replication (414). AM-derived EVs are suggested to play a role in the pathogenesis of acute lung injury by encapsulating TNF-α (415). The majority of studies have been termed EVs “microvesicles” due to their size, but more recently, larger “macrolets” containing IL-6 have been described which are capable of engulfing and killing *E. coli* following macrophage LPS stimulation (416). The full role of *S. aureus-*infected macrophage EVs on host-pathogen interactions and their interplay with the adaptive immune response merits further studies as possible targets for therapeutic approaches.

## Therapeutic Approaches to *S. aureus* Infection

### Antimicrobials

*S. aureus* has acquired resistance to a wide range of antibiotics, which is an expanding problem for the treatment of human infections. In fact, the specter of antimicrobial resistant (AMR) *S. aureus* has been described as a pandemic (417), with global incidence rising (418–421). Resistance most commonly arises due to horizontal gene transfer from resistant bacteria, however, mutation of the *S. aureus* chromosome and mobile genetic elements may also lead to resistance (417, 422). Antibiotic resistance is a particular threat to modern medicine, with multiple procedures dependent on antibiotic use (6). Last resort antibiotics used to treat MRSA are often expensive, less efficacious, and more likely to cause severe side effects (422).

The intracellular nature of *S. aureus* impedes antibiotic activity, as many antibiotics cannot access the intraphagocyte niche (423, 424). Methods to combat this have included development of intracellular antimicrobials (425), nanoparticles which can distribute antimicrobials to infected macrophages (426, 427) and active targeting of macrophages to induce receptor-mediated endocytosis, releasing singlet oxygen to kill intracellular *S. aureus* (428). Furthermore, therapeutic nanoparticles which favored pro-inflammatory macrophage polarization enabled clearance of *S. aureus* biofilms *in vivo* (169). The antimicrobial protein plectasin, can kill *S. aureus* inside THP-1 macrophages *in vitro*, or inside peritoneal macrophages *in vivo*, however, plectasin is significantly more effective against extracellular bacteria (425). A nanogel which preferentially targets macrophages uses bacterial enzymes to initiate release of antibiotic (vancomycin), inhibiting MRSA growth at sites of infection *in vivo* (426). After treatment with the nanogel, zebrafish embryos infected with MRSA survived to significantly higher levels with no visible (GFP-expressing) bacteria 9 h post-infection. Similarly, macrophages treated with nanogel had significantly reduced CFUs recovered from *S. aureus* infected RAW 264.7 macrophages (426). Also, conjugation of penicillin G to squalene enabled antibiotic endocytosis into J774 macrophages, whereby *S. aureus* was significantly less able to survive intracellularly (427).

### Vaccines

Decades of work have been devoted to production of a *S. aureus* vaccine, however, none has yet been approved (14, 15). A key difficulty in producing a *S. aureus* vaccine is that it must provide broad immunity, since the bacteria can cause a wide range of infections in a variety of tissues. *S. aureus* was traditionally believed to be extracellular; however, it is now recognized as a facultative intracellular pathogen. This may partially explain the lack of an effective *S. aureus* vaccine, especially with *S. aureus* able to exist within immune cells. Indeed, since *S. aureus* exploits macrophages during infection, vaccine design inducing successful macrophage defenses against *S. aureus* could be valuable.

Attempts have been made to produce whole cell or live/killed vaccines against *S. aureus*, but these have failed to produce effective immunity (14, 15). Vaccines have been targeted against *S. aureus* polysaccharide, with initial positive results in animal studies and partial protection in early human trials (429, 430). Other targets include surface polysaccharide poly-N-acetylglucosamine (431, 432), surface proteins such as iron surface determinant (Isd) A or IsdB (433, 434), clumping factor (Clf) A or ClfB (435–437) and fibronectin binding protein (FnBP) (438). These vaccines led to partial immune protection, but overall they were not successful (439). A limiting factor may be that the proteins used are not essential components of *S. aureus* (440). To combat this, research groups have tried combining multiple antigens into a single vaccine. Newer approaches include targeting *S. aureus* molecules which stimulate varied immune responses, to mimic the different immune responses observed with natural *S. aureus* infection. It is now thought that approaches which induce Th1/Th17 responses may be more effective, although this is thought to ideally be best when combined with induction of opsonophagocytic antibody generation (439, 441). Many of the aforementioned vaccine studies investigated whether the treatment was able to induce opsonophagocytic killing by phagocytes (432, 433, 437, 438). However, it has also been suggested that vaccines which neutralize *S. aureus* toxins, rather than aiming to induce opsonophagocytic killing, may be more effective (15).

Differences in animal and human responses to vaccines hinders their production. Some studies have found that, despite promising results in animal models, human trials showed no protective immunity (433, 439). This suggests that positive animal trials do not correlate with positive human immune responses, which may be in part to the differences between the human and murine/rabbit immune system. One of the limitations of *S. aureus* mouse models is that a much higher dose of the bacteria is required to initiate infection when compared to the estimated human infective dose. Co-injection of mice with commensal bacteria alongside a dose of *S. aureus* more comparable to natural human infection led to increased CFUs, and decreased survival of the mice (272). This phenomenon was labeled “augmentation.” As *S. aureus* exists in a polymicrobial environment, this model is likely closer to that of natural infection. It is possible that using this augmentation model in murine models to better represent human infection would improve the assessment of therapeutic efficacy against *S. aureus*.

## Future Perspectives

*S. aureus* is a highly successful pathogen due to a wide variety of virulence factors and immune evasion strategies (22). Macrophages play a crucial role in the control of *S. aureus* infection as macrophage depletion in mice led to increased susceptibility to *S. aureus* (61, 272). However, macrophages do not always eliminate staphylococci, which can use the macrophages as a reservoir for persistence, causing continued infection. Therefore, it is important to further characterize the mechanisms used by *S. aureus* to overcome macrophage killing and manipulate the host cell as these may present novel therapeutic adjuncts preventing dissemination and persistence of infection.

It remains unclear which antimicrobial strategy above all others is responsible for killing the majority of *S. aureus*. As detailed above, macrophage killing mechanisms, including ROS, RNS, phagosome acidification, antimicrobial enzymes and AMPs, nutritional immunity and autophagy contribute to *S. aureus* clearance and it is therefore likely through a combination of these mechanisms. NOX2-dependent ROS is seemingly critical, as CGD patients are particularly susceptible to *S. aureus* infection (208). *S. aureus* appears to require exposure to an acidic environment for intracellular survival, again suggesting NOX2-dependent ROS rather than downstream phagosomal maturation is most critical for bacterial killing. Further studies are required to confirm which ROS, within the macrophage phagosome, are necessary to overcome *S. aureus* infection to fully understand the ROS killing capacity. Since NOX2-dependent ROS appears to be vital for bacterial killing, enhancement of macrophage NOX2 activity may be useful as a therapeutic target.

In addition to further characterizing the killing mechanism, a greater understanding of the strategies used by *S. aureus* to evade the host is required to prevent dissemination of infection. To date, much effort has gone into evaluating the role of neutrophils in *S. aureus* infection, while macrophages, despite being a source of bacterial persistence, have been far less studied. The role of macrophages in controlling infection highlights these cells as an important target for investigation and exploitation. Indeed, studies targeting macrophages during *S. aureus* infection show beneficial outcomes (426–428).

Finally, in light of the rising antimicrobial resistance, determining the optimal antibiotic strategies to control *S. aureus* infections, and use of novel agents or combinations to provide synergistic activity merit further studies. The use of immunomodulation and preventative approaches to the peri-operative patient, if fruitful, would lead to significant decreases in the public health burden posed by *S. aureus*.

## Author Contributions

All authors contributed to the writing and editing of the article and GP and JG created the figures. All authors contributed to the article and approved the submitted version.

## Funding

This work was supported by Medical Research Council (MRC) grant MR/R001111/1 and was supported in part by AMR cross-council funding from the MRC to the SHIELD consortium “Optimising Innate Host Defence to Combat Antimicrobial Resistance” MRNO2995X/1.

## Conflict of Interest

The authors declare that the research was conducted in the absence of any commercial or financial relationships that could be construed as a potential conflict of interest.
